# Editorial for the Special Issue on Photonic Chips for Optical Communications

**DOI:** 10.3390/mi15070867

**Published:** 2024-06-30

**Authors:** Jing Xu, Minhao Pu

**Affiliations:** 1 School of Optical and Electronic Information, Huazhong University of Science and Technology, Wuhan 430074, China; 2 Wuhan National Laboratory for Optoelectronics, Huazhong University of Science and Technology, Wuhan 430074, China; 3 DTU Fotonik, Technical University of Denmark, Øresteds Plads 343, 2800 Kongens Lyngby, Denmark

In this era of data explosion, optical communications have endowed the digital world with the capability for high-speed, large-capacity data flow transmission. Integrated photonic chips, leveraging low-loss materials, compact structures, and power-saving components, meet the demands for cost-effectiveness and energy efficiency in applications such as data center interconnections, long-distance information transfer, metro/access networks, and so on. Numerous scholars have devoted their research efforts to enhancing the performance of integrated devices and broadening the scope of their applications in optical communications. This dedication revolves around providing solutions that are both economical and energy efficient. It is evident that photonic chips serve as the backbone of optical communications in various contexts, including all-optical signal processing without the need for optical–electric/electric–optical conversion in optical relays. A wide array of micro and nano-devices, constructed from diverse materials, has been developed to create practical modules such as lasers [1,2], pulse generators [3], modulators [4,5,6], nonlinear devices [7,8,9,10], and fiber-to-chip couplers [11,12,13,14]. These modules play an indispensable role in enabling system-level applications, encompassing but not limited to coherent communications, multiplexing technologies (WDM, SDM, MDM), classic signal processing, and quantum information processing.

The present Special Issue of *Micromachines*, entitled "Photonic Chips for Optical Communications", features a total of ten papers. Six of these papers focus on addressing the main challenges in the field of photonic chips for optical communications, while the remaining four are review papers covering topics such as AlGaAs, femtosecond laser-fabricated chips, slow-light electro-optic modulators, and spectral interferometry with frequency combs. This Special Issue introduces the latest technologies and shows the progression of photonic chip development.

The first group of authors review different aspects of integrated photonic chips, from material platforms, fabrication methods, and functional modules to system applications.

Several materials have emerged as the basis of photonic chips since integrated photonics was pioneered. Mobini et al. carefully reviewed the progress of AlGaAs [15], which serves as an ultra-high nonlinear material supporting all-optical signal processing operations, especially the all-optical wavelength conversion employing four-wave-mixing (FWM). Since the early nonlinear experiments, there has been a continuing quest for new materials and waveguide platforms with optimized nonlinear optical performances. This review compares multiple nonlinear materials with their refractive index, nonlinear coefficients, and typical losses. Among all these nonlinear waveguide materials, III–V semiconductors stand out due to their natural suitability for the monolithic integration of passive and active nonlinear optical devices. They can accommodate passive waveguides for light steering and nonlinear manipulation, laser sources, modulators, and detectors monolithically on the same chip. AlGaAs is one of the III–V integrated nonlinear photonic platforms that has been considered to date, and has shown truly stunning and extraordinary advances in nonlinear optics. It is worth noting that the nonlinear coefficients of AlGaAs waveguides perform diversely with the changes to the elemental composition of Al and Ga, which can be expressed by Al_x_Ga_1−x_As with the variance of x. Different waveguide structures are then introduced. The emphasis on nonlinear effects, including $\chi^{2}$, $\chi^{3}$, and even $\chi^{5}$ nonlinearities, are given and discussed carefully with phase-matching techniques. On the other hand, dispersion and nonlinear absorptions are summarized. The absorption is highly wavelength-dependent due to the relation between photon energy and material bandgap. From the perspective of applications, Kerr nonlinearities and their related functions are listed in detail. Consequently, nonlinear effects in AlGaAs enabled wavelength conversion, frequency combs generation, spectroscopy, and integrated quantum photonics. AlGaAs-OI may represent one of the best solutions for highly efficient nonlinear photonic devices thanks to its low propagation loss and potential for dispersion engineering. These features point to a bright future for AlGaAs monolithic circuits with nonlinear functionalities.

Femtosecond laser writing plays a critical role in chip manufacturing methods. In the second review, Cai et al. presented femtosecond laser fabrication as an acknowledged technique for producing integrated photonic devices [16]. With the development and rising need for three-dimensional waveguides, ultrafast laser technology, encompassing picosecond and femtosecond lasers that have developed rapidly since the 1990s, has been a good candidate for three-dimensional fabrication. Based on femtosecond laser direct writing, several types of waveguides can be manufactured with different morphological structures (single-line, double-line, and depressed cladding waveguides) and dimensions in various materials. Femtosecond laser writing has been applied to several active materials and is able to fabricate active devices for optical communications. Waveguide lasers, amplifiers, electro-optic (EO) modulators, and frequency converters have been investigated. Passive devices such as polarization multiplexers, mode multiplexers, and fan-in/fan-out devices (couplers) are presented. Finally, photonic wire bonding is introduced as a novel concept for the automated 3D fabrication of optical chip-to-chip interconnections. The femtosecond laser fabrication technology has shown a powerful ability and unique capability in constructing diverse waveguide devices with strong qualities for future photonic networks. Future efforts in this research field would primarily focus on exploring undisclosed physical mechanisms, developing new fabrication techniques, and extending the application range. We envision great potential of the femtosecond laser fabrication technology for further exploring emerging or potential photonic functionalities and systems in the near future.

The maturity of manufacturing processes renders large-scale photonic chips possible. However, the development of independent optoelectronic devices is equally of crucial importance. EO modulators play a central role in implementing the electric signal to the optical signal. Slow-light effect-based silicon modulators have attracted attention recently, as they ensure high-speed modulation and large optical bandwidth and feature a small footprint compared to Mach–Zehnder and micro-ring modulators. Han et al. commented on the recent progress of silicon-based slow-light electro-optic modulators towards future communication requirements [17]. Two kinds of structures, photonic crystals as well as waveguide grating, are summarized and compared meticulously. As can be seen in the review, while silicon waveguide grating modulators have lower loss than silicon photonic crystal modulators, silicon photonic crystal modulators are superior in the aspects of transmission rates and transmission format complexity, especially the operating wavelength range, which is far beyond that of silicon waveguide grating modulators. Both of them hold excellent high-speed transmission performance. The modulation efficiency can be further improved, and the footprint can be reduced significantly by harnessing the slow-light effect, demonstrating the potential of high-density optoelectronic integration. With the continuous efforts of researchers, silicon-based slow-light electro-optic modulators will achieve further progress as a core unit device, leading to significant developments in silicon photonics and integrated optical signal processing.

The last review contributes to a concrete application—spectral interferometry with frequency combs [18]. Laser frequency combs formed by evenly spaced optical frequencies whose location in the electromagnetic spectrum can be set with the accuracy provided by atomic frequency references have become enabling tools for precision frequency synthesis and metrology. The most remarkable applications of frequency combs lie in optical spectroscopy. A few techniques in which frequency combs enable or support spectral interferometry are introduced, including time-domain interferometry, spectral-domain interferometry, comb-calibrated swept-wavelength interferometry, Fourier-domain mode-locked lasers for optical coherent tomography, and dual-comb interferometry. Optical arbitrary waveform characterization through spectral slicing is also carried out to achieve single-shot measurements of an arbitrary waveform. Spectral interferometry has been employed in various applications, like molecular spectroscopy, the characterization of optical fibers (extraction of loss, group delay, dispersion), optical coherence tomography to perform noninvasive imaging of biological samples, the analysis of micro photonic devices, and photonic signals. Integrated comb sources are the trends, from mode-locked lasers and electro-optic combs to Kerr microcombs. The tunability of comb parameters such as offset or line spacing can trigger more technological prospects. Overall, the field is broad and rapidly evolving, as the author concluded.

The next section of this Special Issue includes progress in different aspects of integrated chips. High Q silicon micro-ring resonators are essential building blocks for on-chip signal processing. Zeng et al. demonstrated a reflowing photoresist and oxidation smoothing process to fabricate an ultra-high-Q silicon micro-ring with an average Q factor up to 1.2 × 10^6^ [19]. The waveguide loss is as low as 0.27 dB/cm for TE_0_ mode using a 1.5 µm wide rib multimode waveguide. The application of FWM for signal wavelength conversion is then tested using ultra-high-Q micro-ring resonators. The typical conversion efficiency of −17 dB under a 6.5 dBm continuous wave pump is demonstrated. The devices show the potential to improve Q factors and FWM conversion efficiency under low pump power and are helpful in optical communications. Another work introduces high-power soliton microcombs generated with the help of power amplification in Erbium-doped fiber using silicon nitride micro-resonators. Chen et al. presented a combined technique to access power-sufficient optical microcombs with a photonic-integrated soliton microcomb and home-developed erbium-doped gain fiber [20]. The soliton microcomb is generated in an integrated Si_3_N_4_ micro-resonator chip, which serves as a full-wave probing signal for power amplification. The micro-resonators are designed with a radius of 220 µm and have a cross section of 1800 × 780 nm^2^, able to access the whole anomalous dispersion region near 1550 nm to ensure dissipative soliton formation. The solitons generated via the laser tuning scheme are injected into self-made EDF for full-wave amplification. After the amplification, more than 40 comb modes, with 115 GHz spacing, reach the onset power level of >−10 dBm, yielding a peak gain of up to 20 dB. The results show a comparative gain bandwidth at the 10 dB level, and this performance could be even better than the commercial performance at a low current. The combination of chip-scale microcombs and highly efficient EDF amplification can be used to access the power-sufficient and broadband-multi-frequency lasers desired in current WDM systems.

MZ modulators underpin devices for all levels of optical network. Sun et al. proposed a folded heterogeneous integrated silicon and lithium niobate modulator featuring low optical loss, low driven voltage, and a large modulation bandwidth [21]. The unique folding structure significantly reduces the device size by harnessing U-turn optical waveguides consisting of Euler bends and waveguide crosses. The total device length is only 9 mm. The highly efficient modulating function is performed with a half-wave voltage of 1.24 V. The device can support a 128 Gb/s data rate by testing PAM-4 modulations, and a moderate BER, as well as an eye diagram, are carried out. 

Silicon photonic crystal waveguides are also critical integrated components that can be employed as optical filters, modulators, in switching, etc. Pulse propagation in silicon photonic crystal waveguides is affected by the nonlinear properties and the inherent dispersion of the waveguide, which is worth considering in optical signal processing. Wang et al. studied the soliton pulse propagation in a silicon photonic crystal waveguide using the sum frequency generation cross-correlation frequency-resolved optical measurement setup [22]. The soliton pulses exhibited broadening, blue shift, and evident pulse acceleration. The dynamics of the pulses were analyzed by simulating the nonlinear Schrödinger equation and they agreed well with the experimental results. The results conclude that the waveguide length influences the pulse width and shows periodic change with increasing waveguide length. The results help to understand the ultrafast nonlinear behaviors in silicon waveguides and the soliton-based functional elements on CMOS-compatible platforms. 

Topological photonics, with robustness and topological protection features, have attracted much attention recently. Yuan et al. designed a topological wavelength router based on a topology optimization algorithm [23]. The valley photonic crystal, which is an important kind of topological photonic system, is applied to realize on-chip integrated high-performance nanophotonic routers. The optimized router was fabricated on the SOI platform and was experimentally characterized. It supports two channels centered at 1520 nm and 1550 nm, with full widths of the half maximum of 5 nm and 6 nm, respectively. The signal-to-noise ratios of the two transmission peaks are 11.20 dB and 15.76 dB. System robustness is proved by introducing random disorders into the devices. The field distributions disclosed that the external perturbations and defects have very little influence on the functionality of the devices. This work showed that topology-optimized nanophotonic devices were promising in practical applications with high performance and robustness.

The last research work is related to a broadband flat optical frequency comb based on microstructure fiber. Huang et al. proposed a scheme to generate a broadband flat-frequency comb in a cascaded sign-alternated dispersion tellurite microstructure fiber, together with electro-optical modulation [24]. The cascaded sign-alternated dispersion tellurite microstructure fiber benefited from high nonlinearity and controllable dispersion, and can break the bandwidth limit of frequency combs. The author showed that with a seed pulse of 20 GHz repetition rate and a 30 W peak power, an output flat frequency comb covering a 170 nm spectrum could be obtained with good coherence. 

Optical communications assisted by integrated photonic chips are rapidly evolving. Integrated optical devices, such as lasers, all-optical signal processors, wavelength converters, and detectors, are changing the environment of the optical interconnect center. The system performance can be improved by using new materials and new structures and the advances in technologies are helping photonic chips to move rapidly towards practicality.
